# Genetic and geographical structure of boreal plants in their southern range: phylogeography of *Hippuris vulgaris* in China

**DOI:** 10.1186/s12862-016-0603-6

**Published:** 2016-02-09

**Authors:** Qixiang Lu, Jinning Zhu, Dan Yu, Xinwei Xu

**Affiliations:** National Field Station of Freshwater Ecosystem of Liangzi Lake, College of Life Sciences, Wuhan University, Wuhan, P. R. China

**Keywords:** Boreal plants, China, Chloroplast DNA, Ecological niche modeling, *Hippuris vulgaris*, Microsatellites, Phylogeography

## Abstract

**Background:**

Our current understanding of the evolutionary history of boreal and arctic-alpine plants in their southern range in Asia remains relatively poor. Using three cpDNA non-coding regions and nine nuclear microsatellite (nSSR) loci, we examine the phylogeographic pattern in a broad geographic sampling of the boreal plant *Hippuris vulgaris* to infer its dispersal and diversification in China. In addition, the species distributions at the Last Glacial Maximum (LGM) and at present were predicted using ecological niche modeling (ENM).

**Results:**

The cpDNA results revealed two distinct lineages, A and B. A is restricted to Northeast China; B is distributed in Northwest China, the Qinghai-Tibet Plateau (QTP) and North and Northeast (NNE) China; and A and B diverged ca. 1.36 Ma. The nSSR data revealed two genetic clusters corresponding to the two cpDNA lineages and nonreciprocal hybridization with lineage A as the maternal lineage in Northeast China. Cluster B further divided into three subclusters: I, mainly in NNE China and the northeastern border of the QTP; II, in Northwest China and the QTP; and III, on the QTP. ENM predicted a marked range shift on the QTP at the LGM, retreating from the platform to the northeast and southeast edges.

**Conclusions:**

*Hippuris vulgaris* probably diverged into lineages A and B in high latitudes and then immigrated into Northeast China and Northwest China, respectively. Lineage A persisted and diversified in Northeast China. Lineage B reached the QTP during the mid-Pleistocene, diversified in that region due to the influence of climatic oscillations, migrated into Northeast China and subsequently hybridized with lineage A. Our findings give empirical evidence that boreal plants display complex evolutionary history in their southern range in Asia and provide new insights into the evolution of boreal and arctic-alpine plants.

**Electronic supplementary material:**

The online version of this article (doi:10.1186/s12862-016-0603-6) contains supplementary material, which is available to authorized users.

## Background

Climatic oscillations in the Quaternary have greatly affected the distribution and abundance of boreal and arctic species [1]. These species have undergone major range shifts with the advances and retreats of ice sheets. In the past two decades, numerous studies have focused on plant species in boreal regions and/or arctic latitudes and southern mountain ranges, and different phylogeographic patterns amongst plants have been revealed, especially in Europe and North America [2–5]. Some of these studies included samples from mountains in Central Asia have revealed that Central Asia is an important refugium for boreal and arctic plants and colonization from Central Asia to Europe or to Beringia are conceivable [6–10]. However, colonization routes of these species between Central Asia and mountains in low latitudes in Asia need to be further explored. At present, our understanding of the southern range dynamics of boreal and arctic-alpine plants in Asia is based mostly on data from Japan [11–13]. Phylogeographic studies with a broader geographic sampling in Asia are necessary to better elucidate evolutionary histories of boreal and arctic-alpine plants.

China provides suitable habitats for many boreal and arctic-alpine plant species in their southern range, including the Qinghai-Tibetan Plateau (QTP) and mountain ranges in Northwest China and Northeast China [14]. Two putative routes have been suggested for the migration of the flora between the QTP and the arctic regions: one is the “Central Asiatic Highland Corridor” pathway via mountain ranges in Northwest China [15], and another is the “Himalayan-Hengduan Mountain-Qinling-Northeast China” route [16]. We thus hypothesized that similar colonization routes might also be present in a single species of this plant group. In addition, the QTP is considered a center of the development and differentiation of the elements from boreal or Arctic-Tertiary flora since the late Tertiary and Quaternary glacial periods [17]. Similarly, infra-specific lineage divergence in boreal and arctic-alpine plants might also occur in this region.

To infer refugial areas and colonization routes of boreal and arctic-alpine plants in China, we should conduct phylogeographic studies on species with a widespread range covering the QTP and mountains in northeast and northwest China. In this study, we focused on *Hippuris vulgaris* (Plantaginaceae), an aquatic plant of circumboreal distribution. China is the southern range of this species in Asia, and *H. vulgaris* is mainly distributed in the north, northeast, northwest and southwest of China [18]. The plant is rhizomatous perennial and often grows in fresh water at the edges of ponds, lakes and rivers. A recent chloroplast DNA (cpDNA) phylogeography of *H. vulgaris* in the QTP and adjacent areas revealed a mid-to-late Pleistocene split of two lineages on the QTP and possible colonization between the QTP and Northwest China [19], whereas no specific refugia areas were deduced and no samples from Northeast China were included. We hypothesized that colonization between the QTP and Northeast China was likely existed in *H. vulgaris*. In the present study, we used both cpDNA sequences and nuclear microsatellite (nSSR) data to examine the phylogeographic pattern of *H. vulgaris* based on a large-scale sampling covering its distribution range in China. In addition, we inferred the glacial range dynamics of *H. vulgaris* by using ecological niche modeling (ENM; [20]). Our aims were to (1) test whether lineage divergence on the QTP was detected by both cpDNA and nSSR, and (2) deduced potential refugia and putative colonization routes of *H. vulgaris* in China. Investigating these questions was conducive to (3) elucidating the evolutionary history of *H. vulgaris* in its southern range in Asia.

## Methods

### Plant materials

A total of 637 individuals were collected at 91 sites throughout the distribution range of *H. vulgaris* in China, including Northwest China, the QTP, and North and Northeast (NNE) China (Additional file 1). At each site, 1–16 individuals were sampled at intervals of at least 5 m according to the population size. The leaf material of each individual was collected and immediately stored in silica gel. Voucher specimens were deposited in the herbarium of Wuhan University (WH).

### DNA extraction, amplification and sequencing

Total genomic DNA was extracted from silica-dried leaves using the DNA secure Plant Kit (Tiangen Biotech, Beijing, China). Six cpDNA non-coding regions were examined in preliminary experiment in 2012 and three regions, *trn*H-*psb*A [21], *trn*Q-*rps*16 and *rps*16-*trn*K [22], were chosen due to their high polymorphism (Additional file 2). Polymerase chain reaction (PCR) was performed in a volume of 25 μL containing 10–30 ng of genomic DNA, 0.1 μM of each primer, 0.2 mM of each dNTP, 2 mM MgCl_2_ and 0.6 U of ExTaq DNA polymerase (TaKaRa, Dalian, China) under the following conditions: 3 min at 95 °C, followed by 35 cycles of 30 s at 95 °C, 30 s at 55 °C, and 90 s at 72 °C, and then a final 5 min extension at 72 °C. Amplifications were conducted in a Veriti 96-Well Thermal Cycler (Applied Biosystems, Foster City, USA). The PCR products were purified and sequenced by the Beijing Genomic Institute in Wuhan, China.

### Microsatellite loci development and genotyping

We developed nSSR primers using the microsatellite-enriched library method. Detailed methods and processes followed Wu *et al.* [23]. A total of nine nSSR loci were developed for subsequent analysis (Additional file 3). Microsatellites were amplified under the following conditions: 3 min at 95 °C; 35 cycles of 30 s at 95 °C, 30 s at 49–57 °C (Additional file 3), and 1 min at 72 °C; and a final extension at 72 °C for 5 min. PCR products were analyzed on an ABI 3730XL, and genotyping was performed using GeneMapper version 4.0 software (Applied Biosystems).

### Population genetic structure analyses

All cpDNA sequences were aligned using the program MAFFT 6.7 [24]. The three chloroplast fragments were concatenated for subsequent analyses. Identical sequences were collapsed into a single haplotype using DNASP 5.10 [25], and all sequences of different haplotypes were deposited in GenBank (Accession Nos. KT921227- KT921258). A statistical parsimony network was constructed using TCS 1.21 [26], treating sequence gaps as the fifth character state. In the network analysis, gaps of two or more base pairs were re-coded as single-base-pair indels.

Regarding the nSSR data, genetically distinct clusters of 91 populations were identified using an individual-based assignment approach as implemented in STRUCTURE 2.3.4 [27]. Ten independent runs were performed for each K value (K = 1 to 16) with a burn-in period of 20,000 iterations and 100,000 Markov chain Monte Carlo (MCMC) iterations under the admixture model. The best-fit number of clusters was determined based on the ∆K method [28]. The geographical distribution of different cpDNA haplotypes and nSSR clusters identified was mapped using ArcGIS 9.0 (Esri, Redlands, CA, USA). For both cpDNA and nSSR data, analyses of molecular variance (AMOVA) were used to partition genetic variation among and within populations, as implemented in ARLEQUIN 3.1 [29]. The individuals with the same nSSR genotype were removed and only one was kept within a population in genetic analyses.

### Phylogenetic reconstruction and divergence time estimation

The phylogenetic relationships of cpDNA haplotypes were inferred using maximum likelihood (ML) analysis implemented in GARLI 0.951 [30], starting from random trees and using 10,000,000 generations per search. The ML bootstrap support was estimated from 1000 bootstrap replicates in GARLI. Bayesian inference (BI) implemented in MrBayes 3.1.2 [31] was also used for phylogenetic reconstruction. Two independent MCMC analysis runs were conducted simultaneously, beginning with a random tree, with each run including four chains (one cold and three hot). Two million generations were run, with sampling at every 1,000 generations. Chain convergence was checked using Tracer 1.5 [32], and the first 25 % of samples were discarded as burn-in. The K81uf + I substitution model for the ML and BI analyses was identified using the Akaike information criterion (AIC) implemented in ModelTest 3.7 [33]. The divergence times of cpDNA lineages were estimated by using a Bayesian method implemented in BEAST 1.4.7 [34]. An evolutionary rate of 1.52 × 10^−9^ s/s/y [35] for the three combined cpDNA non-coding regions was used to estimate divergence time under an uncorrelated lognormal relaxed clock model. MCMC analysis of 50,000,000 generations was implemented, in which every 1,000 generations were sampled. The first 10 % of generations were discarded as burn-in, and the parameters were checked using Tracer 1.5.

### Demographic analyses

Regarding the cpDNA data, we examined pairwise mismatch distributions to detect historical demographic expansions using ARLEQUIN. The sum of squared deviations (SSD) and Harpending’s raggedness index (Rag) [36] were used to test the goodness of fit under a sudden-expansion model. Their significances were tested using a parametric bootstrap approach with 1,000 replicates [37]. We also performed two powerful tests, Fu’s *F*_S_ test [38] and the *R*_2_ test [39], to detect potential population growth using DNASP.

### Present and past distribution modeling

ENM was used to predict the suitable past (Last Glacial Maximum, LGM) and present climate envelopes for each lineage (see results), as implemented in MAXENT 3.3.3 k [20, 40]. In addition to the 91 sites in this study (Additional file 1), 47 sites from Chen *et al.* [19] and 68 collection records from the Chinese Virtual Herbarium (www.cvh.org.cn) were included. Based on this total of 206 records, we obtained an environmental data set comprising 19 BioClim variables and altitudes from the WORLDCLIM database with resolutions of 2.5’ ([41]; www.worldclim.org). We examined pairwise correlations among the 20 variables and retained seven environmental variables (annual mean temperature, temperature seasonality, temperature annual range, mean temperature of warmest quarter and coldest quarter, precipitation of warmest quarter and coldest quarter) with pairwise Pearson correlation coefficients of r < 0.7 to minimize biased fitting of niche models. A current distribution model was developed using these seven bioclimatic data layers. This model was then projected onto the LGM dataset simulated by the Community Climate System Model v3.0 [42]. We used the default parameters of MAXENT to construct ENMs. The accuracy of each model prediction was evaluated using the area under the (receiver operating characteristic) curve (AUC).

## Results

### Chloroplast DNA haplotype network

**Fig. 1:**
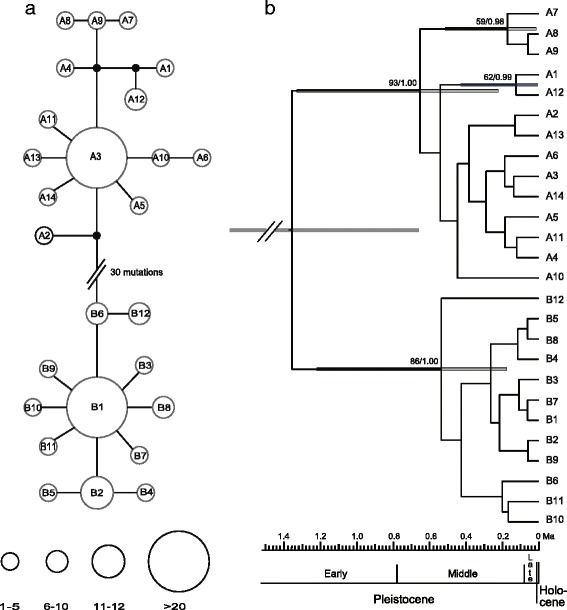
Network and chronogram of 26 cpDNA haplotypes in *Hippuris vulgaris.* **a** Network of genealogical relationships between the 26 cpDNA haplotypes. The black dots represent missing haplotypes. **b** Chronogram of *H. vulgaris* inferred from cpDNA haplotypes using BEAST. Grey boxes indicate 95 % highest posterior density intervals. Numbers at nodes are bootstrap values obtained from ML analysis and Bayesian posterior probabilities in phylogeny reconstruction

**Fig. 2:**
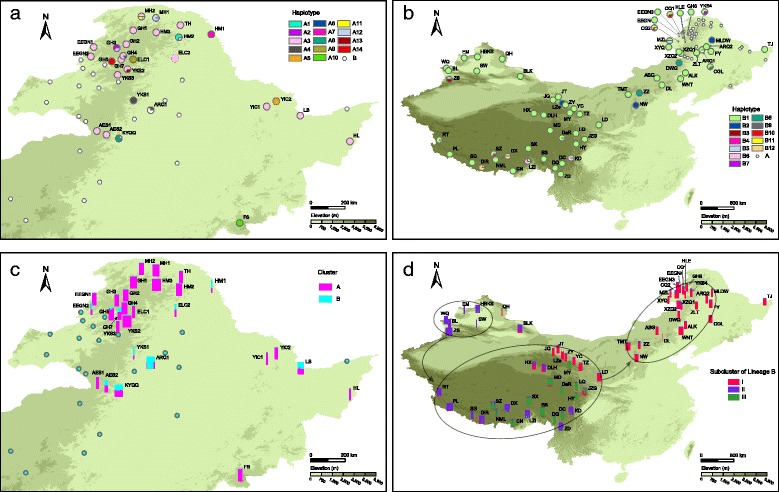
Distribution of cpDNA haplotypes and nSSR clusters in *Hippuris vulgaris* mapped using ArcGIS. **a** Distribution of 14 cpDNA haplotypes in lineage A of *H. vulgaris*. **b** Distribution of 12 cpDNA haplotypes in lineage B of *H. vulgaris*. **c** Distribution of 28 populations of lineage A with nSSR genetic clustering according to the STRUCTURE analysis. **d** Distribution of three nSSR subclusters identified in 63 populations of lineage B using STRUCTURE and putative colonization routes among three geographical regions

The lengths of the aligned sequences for *trn*H-*psb*A, *trn*Q-*rps*16 and *rps*16-*trn*K were 436, 970 and 829 bp, respectively. A total of 56 polymorphic sites, including 21 indels and 35 base substitutions, were observed in the concatenated sequences. The sequences were collapsed into 26 haplotypes (A1-A14 and B1-B12). A haplotype network was constructed with two distinct groups: A and B (Fig. 1a). Group A was restricted to mountainous areas in Northeast China. Group A was found in 28 populations, with haplotype A3 widespread and present in 21 populations, haplotypes A4 and A12 each found in two populations, and other haplotypes each restricted to a single population (Fig. 2a). Group B occurred in all three regions and was found in 64 populations. The most common haplotype, B1, was present in 59 populations; among the remaining 11 haplotypes, only B2 was shared between the QTP and NNE China. Northwest China, the QTP and NNE China contained one, three, and six private haplotypes, respectively (Fig. 2b). Only one population, ARQ1, contained haplotypes from both group A (A4) and group B (B1) (Fig. 2a, b).

### Phylogenetic lineages and divergence time

The trees generated using ML and BI analyses showed the same topology, in which two reciprocally monophyletic lineages (A and B) were recovered with robust support (Fig. 1b). These two lineages corresponded to the two groups in the haplotype network. The divergence time of the two lineages was estimated at 1.36 Ma ago (Ma) (with a 95 % highest posterior density [HPD] interval of 0.66 - 2.52 Ma), which yields time estimates in the early Pleistocene. The diversification of lineages A and B was estimated at 0.65 Ma (95 % HPD: 0.22 - 1.33 Ma) and 0.53 Ma (95 % HPD: 0.17 - 1.22 Ma), respectively (Fig. 1b), both of which are in the mid-Pleistocene.

### Population genetic structure

The STRUCTURE analysis using SSR data suggested K = 2 as the optimal number of genetic clusters based on the calculation of ΔK (Additional file 4). We defined these two clusters as A and B. All individuals of 18 and 63 populations were assigned to cluster A and B with a posterior probability higher than 0.95 (Additional file 5) and had cpDNA lineages A and B, respectively (Fig. 2c, d). Most or all individuals of nine populations were admixed with a probability range from 0.326 to 0.890 (Additional file 5) and had cpDNA lineage A (Fig. 2c). In population ARQ1, two individuals with admixed assignment had cpDNA lineage A, and other individuals with cluster B assignment had cpDNA lineage B (Additional file 5). All of the results suggested that hybridization occurred between lineage A and B and that hybrids were produced only when lineage A was the ovulate parent. Further STRUCTURE analysis was also performed for cluster A and cluster B, and the optimal solution was K = 2 and K = 3, respectively (Additional file 4). In cluster A, all individuals were admixed and no subclusters were identified (Additional file 5). In cluster B, most individuals were assigned to three subclusters with high posterior probability (Additional file 5). These three subclusters had different geographic distributions: subcluster I mainly occurred in NNE China and Northeast QTP edge, subcluster II was mostly found in Northwest China and the QTP, and subcluster III was restricted to the QTP (Fig. 2d).

Hierarchical AMOVA revealed that most of this species’ total variation (92.78 % in cpDNA and 64.02 % in nSSR) occurred among lineages (Table 1), indicating high genetic differentiation between lineage A and B. Nonhierarchical AMOVA for lineage A revealed that most of the genetic variation in both cpDNA and nSSR was distributed within populations (Table 1). For lineage B, the AMOVA results revealed that most of the genetic variation in both cpDNA and nSSR was found within populations, with a low percentage occurring among three regions (Northwest China, the QTP, and NNE China), indicating frequent gene flow among populations and regions (Table 1).

**Table 1:** Analyses of molecular variance based on cpDNA and nSSR data for populations of *Hippuris vulgaris*

Source of variation	cpDNA data		Microsatellite data	
	df	Percentage of total variance (%)	df	Percentage of total variance (%)
All populations				
Among lineages	1	92.78***	1	64.02***
Among populations within lineages	79	2.75***	79	13.33***
Within populations	131	4.47***	523	22.65***
Lineage A				
Among populations	17	36.90*	17	5.76
Within populations	25	63.10***	84	94.24***
Lineage B				
Among populations	62	38.35***	62	40.01***
Within populations	106	61.65***	439	59.99***
Lineage B				
Among regions	2	0.79	2	12.79***
Among populations within regions	60	37.75***	60	30.37***
Within populations	106	61.46***	439	56.84***

Note: * *P* < 0.05, *** *P* < 0.001

### Demographic analyses

Mismatch distributions for lineage A and lineage B were inconsistent with the unimodal curve expected for populations under conditions of demographic expansion (figures not shown). The demographic expansion of lineage A was supported by nonsignificant *H*_Rag_ value but not supported by significant SSD value and Fu’s *F*_S_ and *R*_2_ tests. Similarly, the demographic expansion of lineage B was supported by nonsignificant *H*_Rag_ and SSD values but not supported by Fu’s *F*_S_ and *R*_2_ tests (Table 2). These findings suggest that recent population expansion is not present in either of the two lineages.

**Table 2:** Summary of mismatch distribution parameters and neutrality test for two lineages of *Hippuris vulgaris*

	SSD	*P*	*H*_Rag_	*P*	Fu’s *F*_*S*_	*P*	*R*_2_	*P*
Lineage A	0.257	0.001	0.235	0.983	2.108	0.832	0.053	0.069
Lineage B	0.053	0.067	0.457	0.572	6.791	0.949	0.019	0.104

### Present and past (LGM) distribution of lineages

**Fig. 3:**
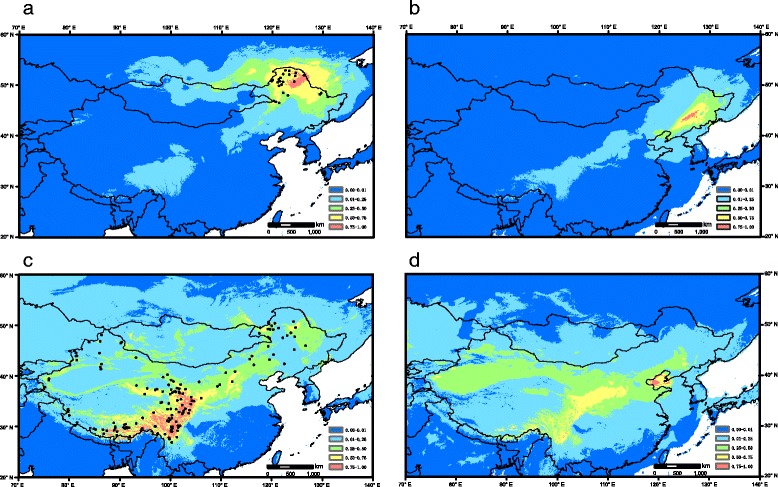
Potential distributions as probability of occurrence for *Hippuris vulgaris* in China mapped using ArcGIS. **a** Potential distributions at the present for lineage A. **b** Potential distributions at the Last Glacial Maximum (LGM) for lineage A. **c** Potential distributions at the present for lineage B. **d** Potential distributions at the LGM for lineage B

For lineages A and B, the AUC values for the ENM were 0.970 and 0.911, respectively, indicating that model prediction performed well. The predicted distribution of lineage A under the current conditions was similar to its actual distribution (Fig. 3a). At the LGM, the potential distribution of lineage A mainly occurred in lowland areas in Northeast China, differing from the present (Fig. 3b). For lineage B, the potential range under the current conditions was generally similar to its actual distribution (Fig. 3c). The predicted distribution at the LGM differed greatly on the QTP, retreating entirely from the platform to the northeast edge and southeast edge (Fig. 3d).

## Discussion

Both cpDNA and nSSR results show that *H. vulgaris* in China is composed of two distinct lineages A and B with different geographic distributions. The distribution ranges of three other species in the genus *Hippuris* are to the north of 53° north latitude, which are in the north of the range of *H. vulgaris* [43, 44]. In addition, the fossil records of *H. vulgaris*, e.g., one of Plio-Pleistocene age from North Greenland [45] and another of middle and upper Pleistocene from central England [46], were also found in the north area of its present distribution. Both species distribution and fossil record suggested that the ancestral range of *H. vulgaris* was in the north of its present range and probably to the north of 53° north latitude. Therefore, lineages A and B had likely split in high latitudes before they migrated into China. The occurrence of different infra-specific lineages in high latitudes of Asia has been revealed in some arctic-alpine plants [3, 5, 6, 8, 9, 47]. Our molecular dating indicates that the divergence of lineages A and B occurred during the early Pleistocene (*ca.* 1.36 Ma), a period displaying minor glacial/interglacial variability before the mid-Pleistocene revolution followed with higher-amplitude fluctuations [48], suggesting that the latitudinal range shifts during the early Pleistocene may not have been sufficient to push *H. vulgaris* southwards into China. The mid-Pleistocene stem ages of lineage A and lineage B (Fig. 1b) also suggest that the two lineages migrated into China driven by the mid-Pleistocene glaciations and thereafter diversified separately. This scenario needs to be tested by further studies including samples from high latitudes of Asia.

Lineage A is restricted to Northeast China, indicating its immigration southwards into this region. The stem age of lineage A in the mid-Pleistocene (*ca.* 0.65 Ma) and the occurrence of its potential distribution in the lowland areas of Northeast China at the LGM (Fig. 3b) suggest that long-standing populations have persisted in Northeast China for several glacial/interglacial cycles. For lineage B, the migration route is more complex. Because the development of a wide arid belt or dryland in northern China and the Republic of Mongolia since *ca*. 2.6 Ma [49, 50] probably acted as a strong physical barrier for aquatic plant dispersal between Northeast China and Northwest China, lineage B should immigrate into China either from Northeast China or from Northwest China. Direct dispersal between the two regions was unlikely, therefore, lineage B reached its present distribution by dispersal either from Northeast China via the QTP to Northwest China or via the reverse route. The former route seems likely because NNE China had the largest number of haplotypes (Fig. 2b), whereas the latter route is supported by the fact that only subcluster I was found in NNE China (Fig. 2d). Combined with the exclusive occupation of the north range of Northeast China by lineage A (Fig. 2a) and the occurrence of inter-lineage hybrids only in Northeast China (Fig. 2c), we considered it more likely that lineage B immigrated southwards into Northwest China, dispersed to the QTP and subsequently migrated into NNE China (Fig. 2d).

The dispersal between highlands in Northwest China and the QTP via the “Central Asiatic Highland Corridor” pathway has been suggested [15] for the migration of the flora between the QTP and the arctic regions. This dispersal pathway was also suggested by Wen *et al.* [51] and verified through the results of a few recent phylogenetic and phylogeographic studies: the same or closely related lineages were present in the two regions [8, 52–54]. As for *H. vulgaris*, its dispersal between Northwest China and the QTP was supported by a shared cpDNA haplotype (Fig. 2b; [19]) and the same genetic subcluster II (Fig. 2d) present in the two regions. This colonization was also in accordance with the migratory route of waterbirds in China [55, 56], which is an important vector for long-distance dispersal of aquatic plants [57, 58]. Furthermore, the fact that *H. vulgaris* is common in the diet of waterbirds at northern latitudes [59] indicates the possibility of long-distance dispersal by waterbirds in *H. vulgaris*. The dispersal between the QTP and Northeast China likely occurred through the “Himalayan-Hengduan Mountain-Qinling-Northeast China” route suggested by Wang [16], based on the geographic distribution of some plants. However, for *H. vulgaris*, another route between Northeast QTP edge and Northeast China seems more likely because the populations from Northeast QTP edge were assigned to genetic subcluster I, the same subcluster as populations from NNE China (Fig. 2d). This route is consistent with the dispersal pathway of a subspecies of sea buckthorn [60].

The QTP has biogeographic significance for alpine plant evolution [51]. Based on evidence from phylogenetic studies on several taxa, Wen *et al.* [51] suggested a pattern of Central Asian origin with subsequent diversification on the QTP. This pattern is suitable for lineage B of *H. vulgaris*. The stem age of lineage B in the mid-Pleistocene (*ca.* 0.53 Ma) is similar to the result of Chen *et al.* [19] (*ca.* 0.48 Ma), estimated based on another cpDNA dataset collected from samples on the QTP, suggesting that *H. vulgaris* reached the QTP during the mid-Pleistocene. Two lineages were revealed on the QTP in Chen *et al.* [19], whereas no cpDNA lineage divergence except three private haplotypes was detected in the present study (Figs. 2b and 3), likely resulting from the use of different fragments. When nSSR data were considered, three subclusters were identified on the QTP: subcluster I in northeast edge, subcluster II from west to east, and subcluster III from middle to east (Fig. 2d). Chen *et al.* found that two cpDNA lineages was overlapped distribution and co-occurred in some populations due to repeated range expansions [19]. This conclusion is confirmed by our nSSR data because the same pattern was also revealed in our populations of subclusters II and III (Fig. 2d) and the sampling of Chen *et al.* [19] did not cover Northeast QTP edge. In addition, we tried to use ENM to infer potential glacial refugia of *H. vulgaris* based on BioClim variables. The distributions of aquatic plants species are correlated with climatic factors [61–63] and occurrences of different genetic lineages are associated with environmental dissimilarity or latitudinal difference in some widespread species [64, 65], indicating that eco-climatological niche modeling based on BioClim variables is applicable for aquatic plants. Based on the prediction of ENM, a marked range shift occurred in *H. vulgaris* at the LGM, retreating from the platform to the northeast edge and southeast edge (Fig. 3c, d). This scenario and the locations of potential glacial refugia have been suggested by many phylogeographic studies on the QTP [66, 67]. The lineage divergence of *H. vulgaris* on the QTP was likely promoted by repeated climatic oscillations during mid-late Pleistocene.

## Conclusions

Our study reveals that *Hippuris vulgaris* is highly structured genetically and geographically in China, with signs of immigration, dispersal, diversification and hybridization. This study provides empirical evidence that boreal plants display complex evolutionary history in their southern range in Asia. Comparative studies on other boreal and arctic-alpine plants would be valuable for better understanding the colonization and diversification in their southern range in Asia.

## Availability of supporting data

The data set supporting the results of this article is included within the article and its additional files. The 26 cpDNA sequences supporting the results of this article are available in the National Center for Biotechnology Information (GenBank) under accession numbers KT921227-KT921258, http://www.ncbi.nlm.nih.gov/Genbank/. Moreover, the nSSR data and datasets for ecological niche modeling are deposited in LabArchives (http://www.labarchives.com/bmc) and are available via the DOI http://dx.doi.org/10.6070/H4K64G3F.

## Additional files

Additional file 1:
**Geographic origins, sample sizes and cpDNA haplotypes of the 91 Hippuris vulgaris populations studied.** (DOCX 28 kb) Additional file 2:
**Polymorphisms in six cpDNA non-coding regions screened in preliminary experiment based on 8 individuals of Hippuris vulgaris.** (DOCX 17 kb) Additional file 3:
**Characteristics of nine nuclear microsatellite markers developed in Hippuris vulgaris.** (DOC 38 kb) Additional file 4:
**Modeling of the numbers of genetic clusters in Hippuris vulgaris for (a) all 91 populations, (b) the 18 populations in lineage A, and (c) the 63 populations in lineage B, respectively, using STRUCTURE.** (DOC 140 kb) Additional file 5:
**The bar plot depicts the STRUCTURE admixture coefficients for individuals of Hippuris vulgaris.** (a) The bar plot for all populations when K = 2. (b) The bar plot for 18 populations of lineage A with no hybrids when K = 2. (c) The bar plot for the 63 populations of lineage B when K = 3. A single vertical bar displays the membership coefficient of each individual, with population names shown on the bottom. (DOCX 325 kb)
