# Supplementary material for: Genetic and geographical structure of boreal plants in their southern range: phylogeography of Hippuris vulgaris in China
Source: BMC Evol Biol. 2016 Feb 9;16:34. doi: 10.1186/s12862-016-0603-6 (PMC4748637; doi:10.1186/s12862-016-0603-6)
Supplement: Additional file 1: — Geographic origins, sample sizes and cpDNA haplotypes of the 91 Hippuris vulgaris populations studied. (DOCX 28 kb) [file 12862_2016_603_MOESM1_ESM.docx]

**Additional file 1.** Geographic origins, sample sizes and cpDNA haplotypes of the 91 *Hippuris vulgaris* populations studied.

| Population name | Voucher | Location | Latitude | Longitude | cpDNA | |  | nSSR | |
| --- | --- | --- | --- | --- | --- | --- | --- | --- | --- |
|  |  |  |  |  | Indiv. | Haplotype |  | Indiv. | Genotype No. |
| DL | Xia11 | Duolun, Neimenggu | 42.1877 | 116.4810 | 2 | B1 |  | 1 | 1 |
| QH | Xia96 | Qinghe, Xinjiang | 46.4927 | 90.2031 | 1 | B1 |  | 1 | 1 |
| HBKS | Xia125 | Hebukesai’er, Xinjiang | 46.8400 | 86.1199 | 3 | B1 |  | 10 | 4 |
| LB | Xu278 | Luobei, Heilongjiang | 47.7100 | 130.9365 | 7 | A3 |  | 7 | 4 |
| MLDW | Xu462 | Molidawa, Neimenggu | 49.5829 | 124.7204 | 4 | B2 |  | 4 | 1 |
| ELC1 | Xu495 | Yakeshi, Neimenggu | 50.6739 | 122.1302 | 3 | A9 |  | 3 | 1 |
| GH4 | Xu501 | Genhe, Neimenggu | 50.7675 | 121.4955 | 7 | A3(6);A6(1) |  | 7 | 5 |
| GH5 | Xu514 | Genhe, Neimenggu | 50.5689 | 121.0489 | 5 | A14 |  | 6 | 2 |
| GH6 | Xu517 | Genhe, Neimenggu | 50.3888 | 120.6345 | 5 | B1 |  | 4 | 4 |
| CQ1 | Xu525 | Chenba’erhu, Neimenggu | 49.9023 | 120.0219 | 5 | B1(3);B10(2) |  | 5 | 5 |
| YKS1 | Xu547 | Yakeshi, Neimenggu | 48.5146 | 122.1538 | 1 | A4 |  | 1 | 1 |
| ARQ1 | Xu556 | Arongqi, Neimenggu | 48.000 | 123.0611 | 6 | A4(2);B1(4) |  | 10 | 3 |
| FY | Xu639 | Fuyu, Heilongjiang | 47.7408 | 124.5492 | 7 | B1 |  | 7 | 3 |
| ZD | Xu2329 | Zhongdian, Yunnan | 27.5047 | 99.8164 | 3 | B1 |  | 12 | 5 |
| DC | Xu2345 | Daocheng, Sichuan | 29.2783 | 100.0831 | 6 | B1 |  | 6 | 1 |
| KD | Xu2349 | Kangding, Sichuan | 30.1636 | 101.498 | 6 | B1(2);B6(4) |  | 12 | 7 |
| HY | Xu2360 | Hongyuan, Sichuan | 32.5035 | 102.3664 | 6 | B1 |  | 6 | 1 |
| JZG | Xu2386 | Jiuzaigou, Sichuan | 33.2582 | 103.7616 | 4 | B1 |  | 12 | 12 |
| DQ | Xu2451 | Deqin, Yunnan | 28.5062 | 98.9115 | 6 | B1 |  | 6 | 1 |
| BS | Xu2456 | Basu, Xizang | 30.0193 | 97.0380 | 6 | B1 |  | 12 | 1 |
| LZi | Xu2470 | Linzi, Xizang | 29.6309 | 94.3825 | 4 | B1(1);B6(3) |  | 12 | 7 |
| DX | Xu2493 | Dangxiong, Xizang | 30.4783 | 91.1002 | 6 | B1(3);B12(3) |  | 12 | 7 |
| DaR | Xu2504 | Dari, Xizang | 33.4886 | 100.0537 | 6 | B1(5);B6(1) |  | 6 | 6 |
| LQ | Xu2515 | Luqu, Gansu | 34.2437 | 102.3340 | 6 | B1 |  | 6 | 1 |
| BLK | Xu2537 | Balikun, Xinjiang | 43.6278 | 93.1856 | 6 | B1 |  | 12 | 9 |
| ABG | Xu2583 | Abaga, Neimenggu | 43.3280 | 115.6771 | 6 | B1 |  | 6 | 3 |
| FS | Xu3802 | Fusong, Jilin | 42.0540 | 127.7497 | 5 | A10 |  | 5 | 1 |
| HL | Xu3881 | Hulin, Heilongjiang | 46.3537 | 133.5564 | 2 | A3 |  | 2 | 1 |
| TJ | Xu3902 | Tongjiang, Heilongjiang | 48.0659 | 133.3989 | 6 | B1 |  | 10 | 2 |
| YiC1 | Xu3976 | Yichun, Heilongjiang | 48.1914 | 129.3485 | 1 | A3 |  | 2 | 1 |
| YiC2 | Xu3977 | Yichun, Heilongjiang | 48.4713 | 129.6679 | 4 | A5 |  | 4 | 1 |
| LZe | Xu4036 | Linze, Gansu | 39.1835 | 100.1357 | 6 | B2 |  | 6 | 2 |
| EM | Xu4256 | E’min, Xinjiang | 46.6655 | 83.9327 | 3 | B1 |  | 3 | 1 |
| WQ | Xu4275 | Wenquan, Xinjiang | 44.9813 | 81.029 | 5 | B1 |  | 9 | 2 |
| BL | Xu4285 | Bole, Xinjiang | 44.5561 | 81.3459 | 6 | B1 |  | 6 | 3 |
| ZS | Xu4315 | Zhaosu, Xinjiang | 43.1572 | 81.4994 | 6 | B1(3);B3(3) |  | 16 | 4 |
| SW | Xu4446 | Sawan, Xinjiang | 44.5139 | 85.9973 | 2 | B1 |  | 2 | 1 |
| HM1 | Xu4519 | Huma, Heilongjiang | 51.9941 | 126.2513 | 1 | A7 |  | 1 | 1 |
| TH | Xu4536 | Tahe, Heilongjiang | 52.4774 | 124.6232 | 4 | A3 |  | 4 | 2 |
| MH1 | Xu4542 | Mohe, Heilongjiang | 52.8605 | 123.3296 | 8 | A3(1);A12(7) |  | 8 | 2 |
| MH2 | Xu4551 | Mohe, Heilongjiang | 52.9393 | 122.5747 | 6 | A3(3);A11(2);A12(1) |  | 6 | 3 |
| GH1 | Xu4552 | Genhe, Neimenggu | 52.2060 | 122.1375 | 6 | A3 |  | 8 | 4 |
| GH2 | Xu4560 | Genhe, Neimenggu | 51.4708 | 121.6171 | 6 | A3 |  | 6 | 3 |
| GH3 | Xu4561 | Genhe, Neimenggu | 51.136 | 121.2697 | 5 | A2(3);A3(2) |  | 5 | 2 |
| EEGN1 | Xu4578 | E’eguna, Neimenggu | 51.0107 | 120.0521 | 5 | A3 |  | 5 | 1 |
| EEGN2 | Xu4582 | E’e’guna, Neimenggu | 50.8277 | 119.9060 | 2 | A3 |  | 2 | 2 |
| EEGN3 | Xu4589 | E’e’guna, Neimenggu | 50.6465 | 119.3794 | 6 | B1 |  | 6 | 6 |
| EEGN4 | Xu4595 | E’e’guna, Neimenggu | 50.2033 | 119.4846 | 5 | B1 |  | 5 | 5 |
| HLE | Xu4601 | Haila’er, Neimenggu | 49.1643 | 120.3329 | 6 | B1 |  | 3 | 2 |
| ARQ2 | Xu4615 | A’rongqi, Neimenggu | 48.5074 | 123.8172 | 5 | B1 |  | 5 | 2 |
| ZLT | Xu4618 | Zhalantun, Neimenggu | 47.5285 | 122.1977 | 6 | B1 |  | 6 | 3 |
| AES1 | Xu4627 | A’esan, Neimenggu | 46.9027 | 120.2542 | 4 | A3 |  | 3 | 1 |
| AES2 | Xu4633 | A’esan, Neimenggu | 46.7067 | 120.7219 | 4 | A3 |  | 4 | 1 |
| MY | Xu5007 | Menyuan, Qinghai | 37.6091 | 101.3198 | 6 | B1 |  | 9 | 2 |
| DLH | Xu5022 | Delingha, Qinghai | 37.2474 | 97.0314 | 6 | B1 |  | 11 | 9 |
| HX | Xu5030 | Haixi, Qinghai | 37.5371 | 95.4274 | 6 | B1 |  | 10 | 7 |
| SX | Xu5058 | Suoxian, Xizang | 31.7348 | 94.4942 | 6 | B1 |  | 12 | 3 |
| MD | Xu5082 | Maduo, Qinghai | 34.7167 | 98.0981 | 6 | B1 |  | 12 | 4 |
| SZ | Xu5103 | Shenzha, Xizang | 30.7542 | 88.7873 | 6 | B1(4);B4(2) |  | 12 | 7 |
| NML | Xu5106 | Nanmulin, Xizang | 29.9981 | 89.0986 | 6 | B1 |  | 5 | 3 |
| DiR | Xu5124 | Dingri, Xizang | 28.5936 | 86.8331 | 6 | B1(1);B12(5) |  | 12 | 4 |
| SG | Xu5127 | Saga, Xizang | 29.4219 | 85.2355 | 6 | B1 |  | 6 | 1 |
| PL | Xu5135 | Pulan, Xizang | 30.8129 | 81.5622 | 6 | B1 |  | 16 | 11 |
| RT | Xu5142 | Ritu, Xizang | 33.1738 | 79.8396 | 6 | B1(5);B6(1) |  | 10 | 4 |
| CN | Xu5177 | Cuona, Xizang | 28.0646 | 91.95 | 5 | B1 |  | 9 | 9 |
| NW | Xu5197 | Ningwu, Shanxi | 38.8729 | 112.2078 | 5 | B2 |  | 10 | 8 |
| YKS2 | Xu6161 | Yakeshi, Neimenggu | 50.1544 | 121.8346 | 3 | A3(2);A13(1) |  | 11 | 6 |
| XZQ1 | Xu6207 | Xinba’erhuzuoqi, Neimenggu | 47.4050 | 119.6194 | 2 | B1 |  | 4 | 1 |
| LD | Xu6568 | Longde, Ningxia | 35.6211 | 106.0972 | 3 | B1 |  | 10 | 2 |
| TZ | Xu6604 | Tianzhu, Gansu | 37.3849 | 102.9608 | 3 | B1 |  | 9 | 4 |
| YC | Xu6613 | Yongchang, Gansu | 38.3009 | 102.0141 | 3 | B1 |  | 6 | 2 |
| ZY | Xu6624 | Zhangye, Gansu | 38.9188 | 100.6105 | 3 | B1 |  | 6 | 3 |
| JQ | Xu6646 | Jiuquan, Gansu | 39.7402 | 98.5502 | 3 | B1 |  | 5 | 2 |
| JT | Xu6669 | Jinta, Gansu | 40.2452 | 99.4315 | 3 | B1 |  | 7 | 2 |
| TMT | Xu6751 | Tumotezuoqi, Neimenggu | 40.6371 | 110.9361 | 3 | B1 |  | 12 | 7 |
| ZZ | Xu6762 | Zhuozi, Neimenggu | 40.8811 | 112.7847 | 3 | B8 |  | 6 | 2 |
| DWQ | Xu6776 | Dongwuqi, Neimenggu | 45.8384 | 119.3241 | 3 | B8 |  | 5 | 2 |
| ALK | Xu6786 | Alukeerqin, Neimenggu | 44.2136 | 120.3678 | 3 | B1 |  | 6 | 3 |
| WNT | Xu6802 | Wongniuteqi, Neimenggu | 43.2299 | 119.5544 | 3 | B1 |  | 11 | 7 |
| CGL | Xu7023 | Chagan lake, Jilin | 45.2035 | 124.4505 | 6 | B1(4);B9(2) |  | 10 | 2 |
| ELC2 | Xu7151 | E’lunchun, Neimenggu | 50.7251 | 124.3319 | 2 | A3(2) |  | 2 | 1 |
| HM2 | Xu7157 | Huma, Heilongjiang | 51.8402 | 124.5425 | 7 | A1(5);A3(2) |  | 7 | 4 |
| HM3 | Xu7167 | Huma, Heilongjiang | 52.0996 | 123.3442 | 10 | A3 |  | 10 | 7 |
| GH7 | Xu7184 | Genhe, Neimenggu | 50.7713 | 121.5031 | 4 | A3 |  | 4 | 1 |
| YKS3 | Xu7189 | Yakeshi, Neimenggu | 49.8597 | 121.3153 | 1 | A3 |  | 4 | 4 |
| YKS4 | Xu7192 | Yakeshi, Neimenggu | 49.4893 | 121.3550 | 3 | B1(2);B7(1) |  | 4 | 2 |
| CQ2 | Xu7196 | Chenba’erhuqi, Neimenggu | 49.2776 | 119.2863 | 5 | B1(3);B10(1);B11(1) |  | 10 | 10 |
| MZL | Xu7200 | Manzhouli, Neimenggu | 49.4930 | 117.7555 | 4 | B1(2);B5(2) |  | 4 | 3 |
| XYQ | Xu7214 | Xinba’erhuyouqi, Neimenggu | 48.4138 | 117.5675 | 2 | B1(2) |  | 3 | 3 |
| XZQ2 | Xu7219 | Xinba’erhuzuoqi, Neimenggu | 47.6733 | 119.2783 | 6 | B1 |  | 10 | 10 |
| KYQQ | Xu7228 | Ke’erqinyouyiqianqi, Neimenggu | 46.5071 | 121.3780 | 6 | A3(1);A8(5) |  | 10 | 2 |
