# Supplementary material for: Genetic and geographical structure of boreal plants in their southern range: phylogeography of Hippuris vulgaris in China
Source: BMC Evol Biol. 2016 Feb 9;16:34. doi: 10.1186/s12862-016-0603-6 (PMC4748637; doi:10.1186/s12862-016-0603-6)
Supplement: Additional file 2: — Polymorphisms in six cpDNA non-coding regions screened in preliminary experiment based on 8 individuals of Hippuris vulgaris. (DOCX 17 kb) [file 12862_2016_603_MOESM2_ESM.docx]

**Additional file 2.** Polymorphisms in six cpDNA non-coding regions screened in preliminary experiment based on 8 individuals of *Hippuris vulgaris*.

| Region | Primer sequence (5′-3′) | Length sequenced  (bp) | Number of  polymorphic sites (%) | Reference |
| --- | --- | --- | --- | --- |
| *trn*H-*psb*A | trnH^(GUG^^)^: CGCGCATGGTGGATTCACAATCC  psbA: GTTATGCATGAACGTAATGCTC | 354 | 7 (1.98%) | [1] |
| *trn*Q-5′*rps*16 | trnQ^(UUG)^: GCGTGGCCAAGYGGTAAGGC  rpS16x1: GTTGCTTTYTACCACATCGTTT | 860 | 16 (1.86%) | [2] |
| 3′*rps*16-5′*trn*K | rpS16x2F2:AAAGTGGGTTTTTATGATCC  trnK^(UUU)^x1:TTAAAAGCCGAGTACTCTACC | 764 | 8 (1.04%) | [2] |
| *trn*L-*trn*F | trnLF_C^(UAA)^: CGAAATCGGTAGACGCTACG  trnLF_F^(GAA)^: ATTTGAACTGGTGACACGAG | 891 | 3 (0.34%) | [1] |
| *trn*G*-trn*S | trnG^(UUC)^: GAATCGAACCCGCATCGTTAG  trnS^(GCU)^: AACTCGTACAACGGATTAGCAATC | 856 | 7 (0.82%) | [2] |
| *rps*16 intron | rpS16_F: AAACGATGTGGTARAAAGCAAC  rpS16_R: AACATCWATTGCAASGATTCGATA | 786 | 8 (1.02%) | [1] |

Reference:

[1] Shaw J, Lickey EB, Beck JT, Farmer SB, Liu W, Miller J, Siripun KC, Winder CT, Schilling EE, Small RL. **The tortoise and the hare II: Relative utility of 21 noncoding chloroplast DNA sequences for phylogenetic analysis.** *Am J Bot*. 2005; **92**:142-166.

[2] Shaw J, Lickey EB, Schilling EE, Small RL. **Comparison of whole chloroplast genome sequences to choose noncoding regions for phylogenetic studies in**

**angiosperms: The tortoise and the hare III.** *Am J Bot*. 2007; **94**:275-288.
