# Supplementary material for: Genetic and geographical structure of boreal plants in their southern range: phylogeography of Hippuris vulgaris in China
Source: BMC Evol Biol. 2016 Feb 9;16:34. doi: 10.1186/s12862-016-0603-6 (PMC4748637; doi:10.1186/s12862-016-0603-6)
Supplement: Additional file 3: — Characteristics of nine nuclear microsatellite markers developed in Hippuris vulgaris. (DOC 38 kb) [file 12862_2016_603_MOESM3_ESM.doc]

**Additional file 3.** Characteristics of nine microsatellite markers developed in *Hippuris vulgaris*.

| Locus | Primer 5’-3’ | Repeat motif | Size range (bp) | Ta (°C) | Accession No. |
| --- | --- | --- | --- | --- | --- |
| Hpv03 | F: CTCCACCATATCTCCTTCACA  R: AAAGCCCAAGCAATACCTAG | (TC)_5_(CA)_8_ | 350-382 | 57 | KT935488 |
| Hpv11 | F: TGACCCTTGCTTCGTTTGT  R: TGGGCATGACTCATTCATAGA | (CA)_9_ | 329-341 | 55 | KT935489 |
| Hpv14 | F: GCATTTATTTGCCTTACTT  R: GCATTTATTTGCCTTACTT | (TG)_7_ | 250-254 | 49 | KT935490 |
| Hpv22 | F: CCTAATCCCTTTCAAACA  R: TAGACAGAAATGTGGAAGAT | (TG)_8_(AG)_5_ | 272-298 | 50 | KT935491 |
| Hpv27 | F: TGTTGGTGGTTCTGATGTT  R: TTCGTATTGCTCCTAAATG | (AGC)_4_(GAT)_5_ | 177-214 | 49 | KT935492 |
| Hpv30 | F: TTGTTCCAGAGTCCAAAAG  R: ACGGAGGTATGAATGAGTTAC | (AC)_8_ | 349-357 | 53 | KT935493 |
| Hpv37 | F: GACCTTTTACATTGCTTG  R: CACCACTGCTTAACTCAT | (TG)_10_ | 372-404 | 50 | KT935494 |
| Hpv67 | F: TTCATTTCTTGCACTTGCT  R: GTTGTCGTCATTGCTTTCC | (AC)_16_ | 284-306 | 51 | KT935495 |
| Hpv75 | F: TTTGTCCGTTGCCATTAGAG  R: TTCGTTTGAAGCCAGAAGAC | (TCA)_10_ | 254-277 | 55 | KT935496 |
