# Supplementary material for: Genetic and geographical structure of boreal plants in their southern range: phylogeography of Hippuris vulgaris in China
Source: BMC Evol Biol. 2016 Feb 9;16:34. doi: 10.1186/s12862-016-0603-6 (PMC4748637; doi:10.1186/s12862-016-0603-6)
Supplement: Additional file 4: — Modeling of the numbers of genetic clusters in Hippuris vulgaris for (a) all 91 populations, (b) the 18 populations in lineage A, and (c) the 63 populations in lineage B, respectively, using STRUCTURE. (DOC 140 kb) [file 12862_2016_603_MOESM4_ESM.doc]

**Additional file 4.** Modeling of the numbers of genetic clusters in *Hippuris vulgaris* for (a) all 91 populations, (b) the 18 populations in lineage A, and (c) the 63 populations in lineage B, respectively, using STRUCTURE.

(a)


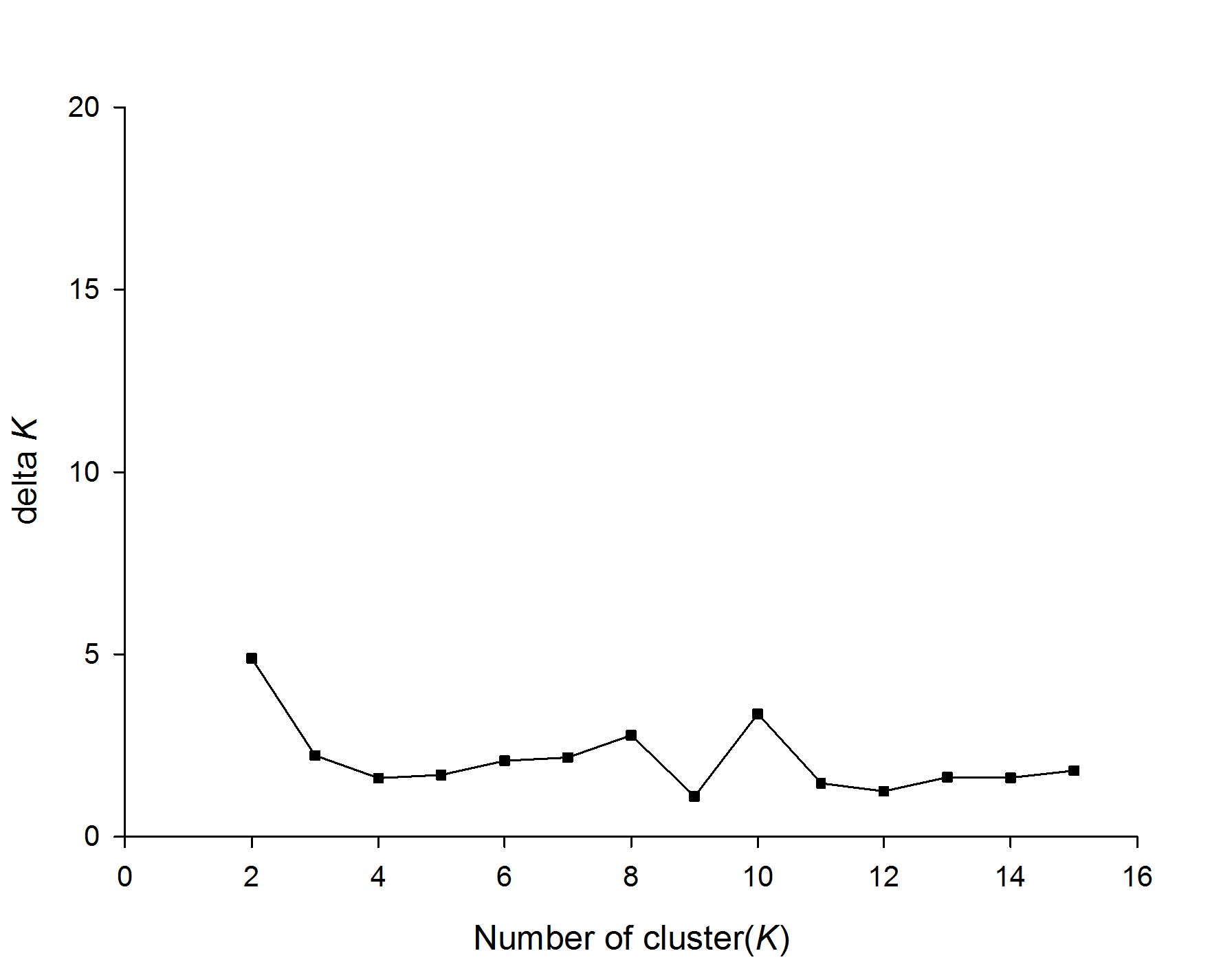


(b)

(c)
