# Supplementary material for: Genetic and geographical structure of boreal plants in their southern range: phylogeography of Hippuris vulgaris in China
Source: BMC Evol Biol. 2016 Feb 9;16:34. doi: 10.1186/s12862-016-0603-6 (PMC4748637; doi:10.1186/s12862-016-0603-6)

**Additional file 5.** The bar plot depicts the STRUCTURE admixture coefficients for individuals of *Hippuris vulgaris*. (a) The bar plot for all populations when K = 2. (b) The bar plot for 18 populations of lineage A with no hybrids when K = 2. (c) The bar plot for the 63 populations of lineage B when K = 3. A single vertical bar displays the membership coefficient of each individual, with population names shown on the bottom.


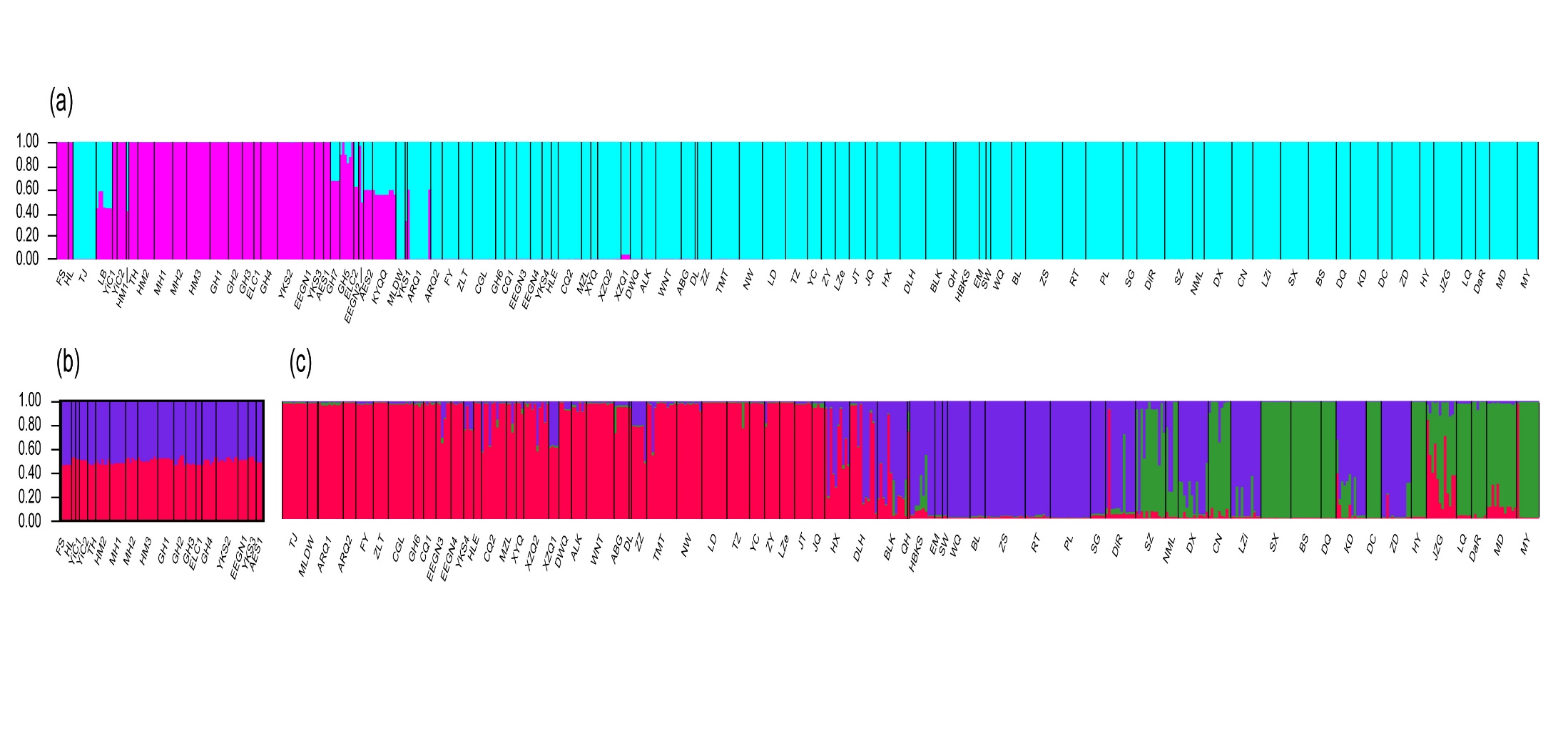

Supplement: Additional file 5: — The bar plot depicts the STRUCTURE admixture coefficients for individuals of Hippuris vulgaris. (a) The bar plot for all populations when K = 2. (b) The bar plot for 18 populations of lineage A with no hybrids when K = 2. (c) The bar plot for the 63 populations of lineage B when K = 3. A single vertical bar displays the membership coefficient of each individual, with population names shown on the bottom. (DOCX 325 kb) [file 12862_2016_603_MOESM5_ESM.docx]
